# New Biomarkers for Patients With Fungal Keratitis From Blood Routine Examination: Neutrophil-to-Lymphocyte Ratio and Platelet-to-Lymphocyte Ratio

**DOI:** 10.1155/joph/5594701

**Published:** 2025-03-11

**Authors:** Aizhen Wang, Menghe Jin, Zhanpeng Yang, Shuaibing Zhou, Juan Yue, Susu Liu, Yanting Xie, Hongmin Zhang

**Affiliations:** ^1^Zhengzhou University People's Hospital, Henan Provincial People's Hospital, Henan Eye Hospital, Henan Eye Institution, Henan Key Laboratory for Ophthalmology and Visual Science, Zhengzhou University, Zhengzhou 450003, Henan, China; ^2^Department of Ophthalmology, Henan University People's Hospital, Zhengzhou 450003, Henan, China

**Keywords:** biomarkers, fungal keratitis disease, neutrophil-to-lymphocyte ratio, platelet-to-lymphocyte ratio

## Abstract

**Purpose:** To assess the potential of neutrophil-to-lymphocyte ratio (NLR) and platelet-to-lymphocyte ratio (PLR) as novel diagnostic and prognostic biomarkers in fungal keratitis (FK).

**Methods:** This study was carried out retrospectively in 77 FK patients and 77 matched cataract controls from Henan Eye Hospital. Peripheral venous blood samples were collected via venipuncture and analyzed using complete blood count for routine clinical evaluation. FK patients were classified into three subgroups: *Fusarium*, *Aspergillus*, and *Candida* groups. Inflammation severity was quantified using standardized clinical scoring. The treatment modalities were used to divide the FK patients into enucleation and nonenucleation groups.

**Results:** NLR and PLR were significantly elevated in FK versus controls (*p* < 0.001). NLR correlated strongly with inflammation scores (*r* = 0.535, *p* < 0.0001), exceeding PLR's moderate correlation (*r* = 0.311, *p*=0.0059). FK patients in the enucleation group had significantly higher NLR (*p*=0.012) and PLR (*p*=0.021) values than those in the nonenucleation group. There were no significant biomarker differences across fungal species (*p* > 0.05).

**Conclusion:** Elevated NLR and PLR values during routine laboratory testing might serve as supplementary indicators for early suspicion of FK and monitoring inflammatory progression, particularly in resource-limited settings where specialized ophthalmic diagnostics are unavailable.

## 1. Introduction

Fungal keratitis (FK) remains a leading cause of corneal blindness in agricultural regions, with *Fusarium*, *Aspergillus*, and *Candida* species accounting for over 80% of culture-proven cases globally [1, 2]. Despite advancements in diagnostic tools such as direct microscopy, corneal scraping, culture, confocal microscopy, and PCR, significant limitations persist in resource-limited rural settings in terms of availability, timeliness, sensitivity, and specificity [3]. As a result, improper diagnosis or delayed treatment is common, which can lead to irreversible complications such as endophthalmitis and even enucleation if not addressed promptly. The diagnostic dilemma is similarly exacerbated by FK's deceptive clinical trajectory (slow onset and subtle symptoms). Without early detection and suitable treatment approaches, the condition becomes debilitating and refractory in its later stages [4]. This diagnostic bottleneck creates urgent demand for rapid, affordable biomarkers that complement existing methods.

Recent studies have explored the use of blood-derived inflammatory biomarkers, particularly the neutrophil-to-lymphocyte ratio (NLR) and platelet-to-lymphocyte ratio (PLR), which have shown promise as indicators of various inflammatory and systemic conditions [5–10]. Emerging evidence positions NLR and PLR as potential diagnostic adjuncts across ocular disorders. Moreover, NLR and PLR levels have also been linked to heightened inflammatory activity, disease severity, and poor prognosis [11–16]. However, the role of NLR and PLR in FK remains underexplored.

This study aims to investigate the potential association between FK and NLR/PLR. By evaluating their levels in FK patients, we seek to understand whether these biomarkers could serve as early indicators for diagnosing FK and predicting its progression, offering a simple, cost-effective tool for stratified FK management in resource-limited settings. Ultimately, this could improve the prognosis and treatment outcomes for FK patients by enabling timely intervention.

## 2. Material and Method

### 2.1. Study Design and Patients

The research was designed as a retrospective observational study, with inherent limitations including selection bias and confounding factors from single-center sampling. The study was approved by the Institutional Review Board (IRB) of Henan Eye Hospital (approval number HNEEC-202419) and adhered to the ethical guidelines of the Declaration of Helsinki. Informed consent was obtained from all included participants.

This study analyzed the medical records of 255 FK patients admitted to Henan Eye Hospital from January 1, 2019, to December 31, 2023. Initially, patients diagnosed with FK based on WHO diagnostic criteria were included, while a control group was selected from patients receiving preoperative screening in the cataract department. General demographic data, such as age, sex, and body mass index (BMI), were collected.

Exclusion criteria included age under 18 years, presence of autoimmune or inflammatory systemic or ocular diseases (except for FK), ocular surgery or trauma within the past 12 months, permanent traumatic eye injury, use of medications that affect blood counts (except for antifungal drugs used in FK treatment), history of malignancy or hematological disorders, pregnancy, and habitual smoking. In addition, patients with incomplete medical records or who did not consent to participate were excluded.

Following the eligibility review, 77 FK patients were included in the study. The control group comprised 77 age- and gender-matched cataract patients without other comorbidities.

### 2.2. Clinical Evaluation

From the medical records, various data were collected, including anterior segment photos, fungal culture results, slit-lamp inspection images, and details of the treatment procedures. Following fungal culture-based identification, in which corneal scrapings were inoculated onto Sabouraud dextrose agar (SDA) using a C-shaped streaking method, FK patients were classified into three etiological subgroups: *Fusarium*, *Aspergillus*, and *Candida* [17]. Inflammation levels in the patients were visually scored using anterior segment photos and slit-lamp inspection images (the two methods complemented each other; in case of any conflict, the slit-lamp results took precedence). The inflammation scoring system, which evaluated area of opacity, opacity density, and surface regularity, was specifically performed by one experienced clinician to ensure consistent and accurate evaluations across multiple time points [18]. Each criterion was graded on a scale from 0 to 4, with higher scores indicating greater severity. In cases where empyema and/or hypopyon were present in the anterior chamber, an additional point was added to the score for that eye. A completely clear, nonscarring cornea received a score of 0 in each category. The final inflammation score for each eye was the sum of the scores across all three categories. More detailed scoring criteria are shown in Table 1. The treatment modalities were used to divide the FK patients into enucleation and nonenucleation groups. Such therapies as pharmacotherapy, penetrating keratoplasty (PK), and anterior lamellar keratoplasty (DALK) were included in the group of nonenucleation.

### 2.3. Blood Sampling

For all participants, 3–5 mL venous blood samples were collected from the antecubital vein in the early morning and mixed with an anticoagulant (EDTA). Complete blood count (CBC) measurements were performed within 2 h using an automated blood cell analyzer (XN9100; Sysmex, Kobe, Japan). The parameters of neutrophil, platelet, and lymphocyte were recorded. The NLR and PLR were calculated by determining the ratio of neutrophils to lymphocytes and platelets to lymphocytes, respectively. The selection of thresholds was determined by maximizing the Youden Index, prioritizing the balance between sensitivity and specificity to ensure optimal diagnostic performance.

### 2.4. Statistical Analysis

The normality of each continuous variable was tested using the Kolmogorov–Smirnov normality test. Continuous variables with a normal distribution were expressed as means ± standard deviations, while continuous variables without a normal distribution were expressed as medians and interquartile ranges. An independent *t*-test, Mann–Whitney *U* test, or chi-square test were used to compare continuous and categorical variables between two groups. For multiple groups, the Kruskal–Wallis test was applied to compare parameters across all groups. Spearman correlation was used to analyze the correlations between inflammatory degree and NLR or PLR. The area under the receiver operating characteristic (ROC) curve (AUC), specificity, sensitivity, and cutoff values were used to determine the accuracy of the NLR and PLR for distinguishing between FK and NFK. Statistical significance was set at *p* < 0.05. The statistical analysis was conducted using the Statistical Package for Social Science 27.0 software (SPSS, Chicago, IL, USA).

## 3. Results

### 3.1. Patient Characteristics

The mean age was 59.43 ± 8.73 years old (range: 35–76) in the FK group and 59.51 ± 8.54 years old (range: 37–75) in the control group (*p*=0.956). The mean BMI was 23.99 ± 3.13 in the FK group and 24.97 ± 3.66 in the control group. Males comprised 44% of both the FK and control groups (*p*=1). There were no significant differences between the groups in terms of age, BMI, and gender distribution (*p*=0.956, *p*=1, and *p*=0.076, respectively).

### 3.2. Parameters of the Blood Test

**Figure 1:**
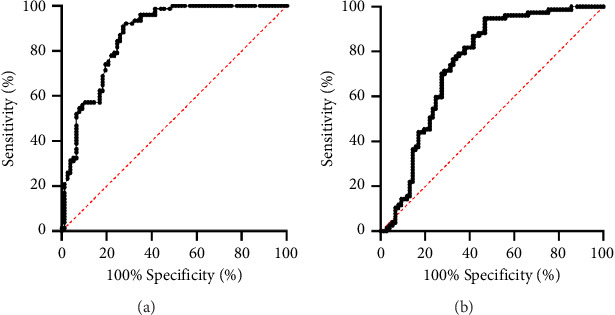
Receiver operating characteristic curves of NLR and PLR for FK predictors. (a) Receiver operating characteristic curves of NLR for FK predictors; area under the curve for the NLR was 0.871 with a cut-off value of 2.35. (b) Receiver operating characteristic curves of PLR for FK predictors; area under the curve for the PLR was 0.749 with a cutoff value of 176. NLR, neutrophil-to-lymphocyte ratio; PLR, platelet-to-lymphocyte ratio. FK, fungal keratitis.

Regarding hematological parameters, neutrophil counts, platelet counts, NLR, and PLR (for all *p* < 0.001) were significantly higher in FK patients compared with control groups. The statistically lower value was observed only in the case of lymphocyte counts (*p*=0.001) between the study and control groups. Comparable of the control and patient groups are summarized in Table 2. The ROC analysis of the evaluated factors is graphically presented in Figure 1. According to the figure, the NLR AUC value that was used to discriminate subjects in the FK and control groups was 0.871. When the best cutoff value was identified as 2.35, sensitivity and specificity values were found to be 71.4% and 92.0%, respectively. The PLR AUC value that was used to discriminate subjects in the FK and control group was 0.749. When the best cutoff value was identified as 176, sensitivity and specificity values were found to be 53.2% and 94.7%, respectively.

Analysis using Kruskal–Wallis H revealed that the median value of NLR was 3.14 in the *Fusarium* group, 2.89 in the *Aspergillus* group, and 2.30 in the *Candida* group. The median value of PLR was 124.23 in the *Fusarium* group, 177.69 in the *Aspergillus* group, and 140.88 in the *Candida* group. There were no statistically significant differences in NLR (*p*=0.534) and PLR (*p*=0.476) noticed among the subgroups (Table 3).

**Figure 2:**
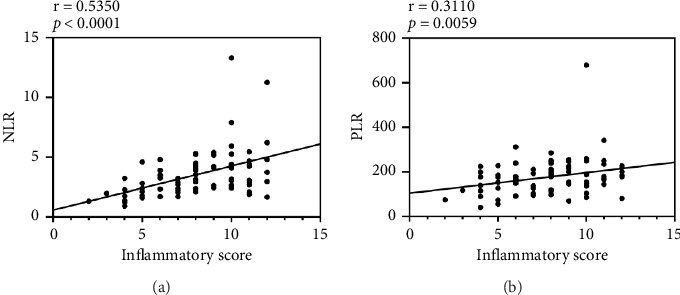
Correlation between NLR/PLR and inflammation score. Spearman correlations were performed for correlation analyses. *p* values less than 0.05 were considered to be statistically significant. (a) Correlation between NLR and inflammation score (*r* = 0.535, *p* < 0.0001). (b) Correlation between PLR and inflammation score (*r* = 0.311, *p*=0.0059). NLR, neutrophil-to-lymphocyte ratio; PLR, platelet-to-lymphocyte ratio.

To investigate the correlation of clinical scoring on CBC-derived inflammatory markers in FK patients, a correlation between inflammation scores and hematological parameters was performed. Spearman correlation analysis in Figure 2 suggested there was a positive relationship between inflammatory scores and NLR (*r* = 0.535, *p* < 0.0001) and PLR (*r* = 0.311, *p*=0.0059).

In two different treatment groups, the median value of NLR value was 3.07 in the enucleation group and 1.98 in the nonenucleation group. The median value of PLR was 179.14 in the enucleation group and 139.07 in the nonenucleation group. The intergroup differences in the median value of NLR and PLR turned out to be statistically significant between the groups (*p*=0.012 and *p*=0.021, respectively). Also, higher NLR and PLR values were observed in the enucleation group (Table 4).

## 4. Discussion

FK represents a severe corneal suppurative inflammation governed by dynamic interactions between fungal virulence and host inflammatory responses. While fungal invasion initiates tissue damage, accumulating evidence suggests that excessive neutrophil activation constitutes a critical driver of terminal-stage corneal opacification and vision loss. Neutrophils, activated by pathogen recognition receptors, release proinflammatory cytokines, neutrophil extracellular traps (NETs), and tissue-damaging molecules such as reactive oxygen species (ROS), which exacerbate inflammation and aid in fungal clearance. However, excessive neutrophil activation can lead to uncontrolled inflammation, further contributing to corneal scarring and depriving patients of the opportunity for corneal transplantation surgery [19–22]. Platelets, traditionally known for their role in hemostasis, are increasingly recognized for their involvement in immunity and inflammation. They possess a dual nature in immune responses, capable of both enhancing inflammatory responses and modulating their strength and temporal dynamics [23]. Moreover, the decrease in lymphocytes, indicative of depressed cell-mediated immunity, has been extensively addressed [24]. Elevated NLR/PLR typically indicates excessive activation of neutrophils and platelets and a relative reduction in lymphocytes, reflecting an exacerbation of the inflammatory response and a disruption of immune balance.

Our study identified NLR (> 2.35) and PLR (> 176) as diagnostic markers for FK, with both parameters significantly elevated compared with controls (*p* < 0.001). While NLR/PLR have demonstrated utility in systemic infections [9, 25], this study provides the first validation in corneal infections, addressing a critical gap in ocular immunology. The NLR threshold, while lower than those in systemic chronic conditions (e.g., COPD NLR > 3.4–16.8; hypertrophic cardiomyopathy NLR > 3.43), aligns with acute ocular pathologies such as retinal artery occlusion (NLR > 1.62) and high myopia (NLR > 2.68) [12, 14, 26, 27]. The blood-eye barrier likely attenuates peripheral immune cell infiltration, reducing systemic biomarker sensitivity compared with nonocular infections. In contrast to chronic systemic inflammation, acute corneal infections may induce different hematopoietic dynamics. Notably, the observed NLR dichotomy—higher than noninflammatory ocular conditions but lower than uveitis (NLR > 5.608)—may result from the former is an intraocular inflammation, whereas FK affects the cornea, leading to differential impacts on the blood-ocular barrier [13]. However, the PLR threshold was found to be elevated relative to other ocular diseases in the current study, though the precise mechanistic drivers of this divergence remain to be fully elucidated. Moreover, even for the same disease, NLR/PLR thresholds vary substantially across different studies [16]. Although a precise cutoff value has not been found yet, its role as a flag of immune system homeostasis was well established.

The prognostic utility of NLR/PLR was reinforced by their significant correlations with both inflammatory severity and surgical outcomes. Mirroring findings in open globe injuries [15] and dry eye disease [28], our data demonstrate these biomarkers' potential for predicting FK progression, with elevated levels indicating higher enucleation risk. Consistent with the previous results, NLR apparently outperformed PLR in diagnostic accuracy and prognostic value, possibly reflecting neutrophils' central role in fungal defense versus platelets' secondary mediator functions. Importantly, both markers showed limited discriminatory capacity for specific fungal species. This observation may indicate either conserved inflammatory responses across pathogens or insufficient statistical power in subgroup analyses due to sample size constraints.

In primary hospitals or resource-limited areas, routine blood tests are easily accessible. While conventional methods like corneal scraping cultures are time-consuming and delay diagnosis, NLR and PLR may provide a rapid alternative as early warning indicators in the absence of efficient etiological tools. Dynamic monitoring of NLR and PLR may help gain valuable insights into the disease's inflammatory status, enabling prompt intervention. In addition, these markers can be used to identify patients who are at high risk for poor outcomes, such as severe tissue damage or the need for surgical interventions like enucleation. Their ability to guide personalized treatment strategies could significantly improve patient prognosis and reduce the long-term impact of FK. The “systemic-to-local” research approach offers new perspectives for the diagnosis and treatment of infectious eye diseases.

Our study also had several limitations. Its retrospective, single-center design and the small number of patients included risked selection bias. Second, single-timepoint measurements preclude tracking dynamic biomarker changes. Third, a huge difference in the disease course may have also affected these risk factors. Lastly, elderly skewed cohort may not generalize to younger populations. Therefore, more stable results would be expected to be gained adequately by clinical trials with larger sample sizes, in which data can be analyzed prospectively.

In conclusion, NLR and PLR might be valuable supplementary indicators for not only early detection but also for monitoring disease progression and tailoring therapeutic approaches in clinical practice. Further research is required to validate these findings and establish their clinical applicability.

## Figures and Tables

**Table 1:** Inflammation scoring system for fungal keratitis patients.

	Grade 1	Grade 2	Grade 3	Grade 4
Area of corneal opacity	1%–25%	26%–50%	51%–75%	76%–100%
Density of corneal opacity	Slight cloudiness, outline of iris and pupil discernable	Cloudy, but outline of iris and pupil remain visible	Cloudy, opacity not uniform	Uniform opacity
Surface regularity	Slight surface irregularity	Rough surface, some swelling	Significant swelling, crater or serious descemetocele formation	Perforation or descemetocele

*Note:* Each criterion (area of opacity, density of opacity, and surface regularity) was graded on a scale of 1–4. A completely clear, nonscarring cornea received a score of 0 in each category. An additional point is added to the total score if empyema and/or hypopyon is present in the anterior chamber. The total inflammation score was the sum of scores from all three categories.

**Table 2:** Comparison between the FK and control groups.

Variables	FK group (*n* = 77)	Control group (*n* = 77)	*p* value
Age (years)⁣^∗^	59.43 ± 8.73	59.51 ± 8.54	0.956
Sex M/F (%)	56/44	56/44	1
BMI (kg/m^2^)⁣^∗^	23.99 ± 3.13	24.97 ± 3.66	0.076
Neutrophil (1/μL)^#^	5.03 (3.86–6.47)	2.96 (2.53–3.60)	< 0.001
Platelet (1/μL)^#^	265.00 (217.00–331.50)	222.00 (203.00–261.00)	< 0.001
Lymphocyte (1/μL)^#^	1.55 (1.35–2.01)	1.97 (1.52–2.24)	0.002
MPV (fL)^#^	10.00 (9.20–11.25)	10.40 (9.80–10.90)	0.116
NLR^#^	3.07 (2.18–4.36)	1.58 (1.27–2.09)	< 0.001
PLR^#^	177.91 (126.81–217.97)	120.86 (98.10–141.81)	< 0.001

*Note:p* value < 0.05 was statistically significant.

Abbreviations: BMI, body mass index; FK, fungal keratitis; M/F, male/female; MPV, mean platelet volume; NLR, neutrophil-to-lymphocyte ratio; PLR, platelet-to-lymphocyte ratio.

⁣^∗^Mean ± SD.

^#^Median (25–75 percentiles).

**Table 3:** Comparison of NLR and PLR values in three fungal species groups.

Variables	*Fusarium* group (*n* = 33)	*Aspergillus* group (*n* = 19)	*Candida* group (*n* = 19)	*p* value
Age (years)⁣^∗^	58.12 ± 6.73	61.42 ± 7.08	59.36 ± 4.49	0.201
Sex M/F (%)	49/51	47/53	58/42	0.762
BMI (kg/m^2^)⁣^∗^	23.69 ± 3.61	24.80 ± 2.36	24.00 ± 2.59	0.450
NLR^#^	3.14 (2.42–4.16)	2.89 (2.11–4.33)	2.30 (1.77–4.55)	0.543
PLR^#^	171.34 (124.23–216.52)	177.69 (98.94–211.57)	140.88 (111.25–187.74)	0.476

*Note:p* value < 0.05 was statistically significant.

Abbreviations: BMI, body mass index; M/F, male/female; NLR, neutrophil-to-lymphocyte ratio; PLR, platelet-to-lymphocyte ratio.

⁣^∗^Mean ± SD.

^#^Median (25–75 percentiles).

**Table 4:** The comparation of parameters of blood test in evisceration and nonevisceration groups.

Variables	Enucleation (*n* = 25)	Nonenucleation (*n* = 25)	*p* value
Age (years)⁣^∗^	62.24 ± 9.07	62.36 ± 8.37	0.961
Sex M/F (%)	44/56	44/56	1
BMI (kg/m^2^)⁣^∗^	24.04 ± 2.81	23.52 ± 3.10	0.542
NLR^#^	3.07 (2.64–4.54)	2.23 (1.66–3.35)	0.012
PLR^#^	179.14 (146.54–230.44)	139.07 (114.79–181.81)	0.021

*Note:p* value < 0.05 was statistically significant.

Abbreviations: BMI, body mass index; M/F, male/female; NLR, neutrophil-to-lymphocyte ratio; PLR, platelet-to-lymphocyte ratio.

⁣^∗^Mean ± SD.

^#^Median (25–75 percentiles).

## Data Availability

The data used to support the findings of this study are included within the article.
