# Corrigendum to “New Biomarkers for Patients With Fungal Keratitis From Blood Routine Examination: Neutrophil-to-Lymphocyte Ratio and Platelet-to-Lymphocyte Ratio”

**DOI:** 10.1155/joph/9875061

**Published:** 2025-08-19

A. Wang, M. Jin, Z. Yang, et al., “New Biomarkers for Patients With Fungal Keratitis From Blood Routine Examination: Neutrophil-to-Lymphocyte Ratio and Platelet-to-Lymphocyte Ratio,” *Journal of Ophthalmology* 2025 (2025): 5594701, https://doi.org/10.1155/joph/5594701.

In the article titled “New Biomarkers for Patients With Fungal Keratitis From Blood Routine Examination: Neutrophil-to-Lymphocyte Ratio and Platelet-to-Lymphocyte Ratio,” there was an error in Table 4 caption. This error is shown below:

“The comparation of parameters of blood test in evisceration and nonevisceration groups” should read “The comparison of parameters of blood test in enucleation and nonenucleation groups”.

We apologize for this error.

